# Tough, Stretchable, and Thermoresponsive Smart Hydrogels

**DOI:** 10.3390/gels9090695

**Published:** 2023-08-28

**Authors:** Yi Luo, Werner Pauer, Gerrit A. Luinstra

**Affiliations:** Institut für Technische und Makromolekulare Chemie, Universität Hamburg, 20146 Hamburg, Germany; yi.luo@chemie.uni-hamburg.de (Y.L.); werner.pauer@uni-hamburg.de (W.P.)

**Keywords:** PVA hydrogel, tannic acid, triple network, self-healing, thixotropy

## Abstract

Self-healing, thermoresponsive hydrogels with a triple network (TN) were obtained by copolymerizing N-isopropyl acryl amide (NiPAAm) with polyvinyl alkohol (PVA) functionalized with methacrylic acid and N,N′-methylene bis(acryl amide) crosslinker in the presence of low amounts (<1 wt.%) of tannic acid (TA). The final gels were obtained by crystalizing the PVA in a freeze-thaw procedure. XRD, DCS, and SEM imaging indicate that the crystallinity is lower and the size of the PVA crystals is smaller at higher TA concentrations. A gel with 0.5 wt.% TA has an elongation at a break of 880% at a tension of 1.39 MPa. It has the best self-healing efficiency of 81% after cutting and losing the chemical network. Step-sweep strain experiments show that the gel has thixotropic properties, which are related to the TA/PVA part of the triple network. The low amount of TA leaves the gel with good thermal responsiveness (equilibrium swelling ratio of 13.3). Swelling-deswelling loop tests show enhanced dimensional robustness of the hydrogel, with a substantial constancy after two cycles.

## 1. Introduction

Smart materials have attracted widespread interest from industrial and academic areas in the past few decades [1]. Intelligent hydrogels, such as hydrophilic, flexible three-dimensional structure materials, exhibit the ability to respond to external stimuli, including light [2,3], temperature [4,5], pH value [6,7], electric potential [8,9], magnetic field [10,11], and so on [12]. The “smart behavior” opens high-tech applications in various fields, such as parts in soft robotics [13], intelligent medical devices [14,15], sensors [16], and biomimetic materials [17,18]. The enhancement of mechanical integrity has long been a challenge to improving typically fragile polymeric hydrogels [19]. In our previous work, a DN (double network) system was designed. (PVA-MA)-g-PNIPAAm thermoset intermediate, and the crystallization of the PVA network enabled a strengthened and thermoresponsive hydrogel. The mechanical robustness of the hydrogel is still apt for improvement in its envisioned application as a thermovalve [20]. The swelling-deswelling loops show a noticeable change in length [21]. Much effort is now devoted to prolonging the service life of such a hydrogel. It remained a challenge to extend the properties of the network and push the limits of “smart” applications. 

The requirements for a useful stimuli-responsive hydrogel for keeping its functionality in valve applications comprise (at least) more or less constant dimensions in swelling-deswelling cycles and a more or less constant and good mechanical strength. The introduction of self-healing properties seems favorable for the latter and, in certain cases, may also be useful for reaching higher dimensional stability [22]. Semi-crystalline PVA has some shape-memory qualities resulting from the position of the crystallites as physical crosslinks [23]. Further fixation in these positions was thought to be advantageous for a functional valve. 

Self-healing hydrogels with a typical soft-and-wet feature, as one of the most promising topics, have recently come into focus [22,24]. They show a unique ability to undergo network self-reform from incidental damage [25]. Self-healing hydrogels exhibit significant advantages for the resetting of their original shape and performance. Self-healing behavior generally originates from dynamic groups, which are classified into two categories, nonautonomic and autonomic [24]. Nonautonomic self-healing hydrogels with covalent bonds (Diels–Alder [26,27], borate bonds [28,29], disulfide bonds [30,31], etc.) need to be triggered by external stimuli, whereas autonomic self-healing gels with noncovalent interactions (H-bonding [32,33], metal-ligand assembles [34,35], host-guest interactions [36,37], electrostatic interactions [37], and the like) have an “unsolicited” action in reassembling themselves under relevant (i.e., usual mild) conditions. A further advantage is their simplicity of fabrication. 

Accordingly, tannic acid (TA) was considered a component for improving the performance of the previously developed responsive hydrogel. It is a natural water-soluble polyphenol compound abundant in pyrogallol and catechol groups (25 phenolic hydroxyl groups in one molecule) [38]. This special molecule structure exhibits extensive opportunities for forming and reforming reversible H bonds, which makes it an ideal ingredient for multifunctional self-healing hydrogels. It has been found that TA complexes with neutral polymers with hydrogen bonding sites, such as PNIPAAm [39,40] and PVA [41,42]. The combination of substantial amounts (>10 wt.%) and PVA indeed has led to substantial strong hydrogels and with shape-memory behavior [43,44,45,46,47]. 

Here, we present a triple-network thermosensitive network with enhanced mechanics over comparable double-network hydrogels. TA was introduced to the system for its multifunctionality. Thus, a tough, thermoresponsive, self-healing PVA/TA/PVA-MA-g-PNIPAAm triple network hydrogel was obtained. Plenty of reversible hydrogen bonds (H-bonds) are possible between TA and the PVA, which contribute to the good self-healing efficiency (81%) of the hydrogel and assist in keeping the initial dimensions. 

## 2. Results and Discussion

### 2.1. Formation Pathway and Structural Analysis of PVA/TA/PVA-MA-g-PNIPAAm Triple Network Hydrogels

**Scheme 1:**
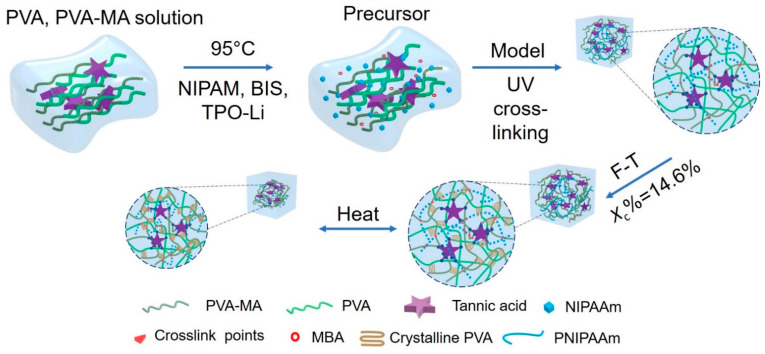
The forming of the triple-network hydrogels.

**Figure 1:**
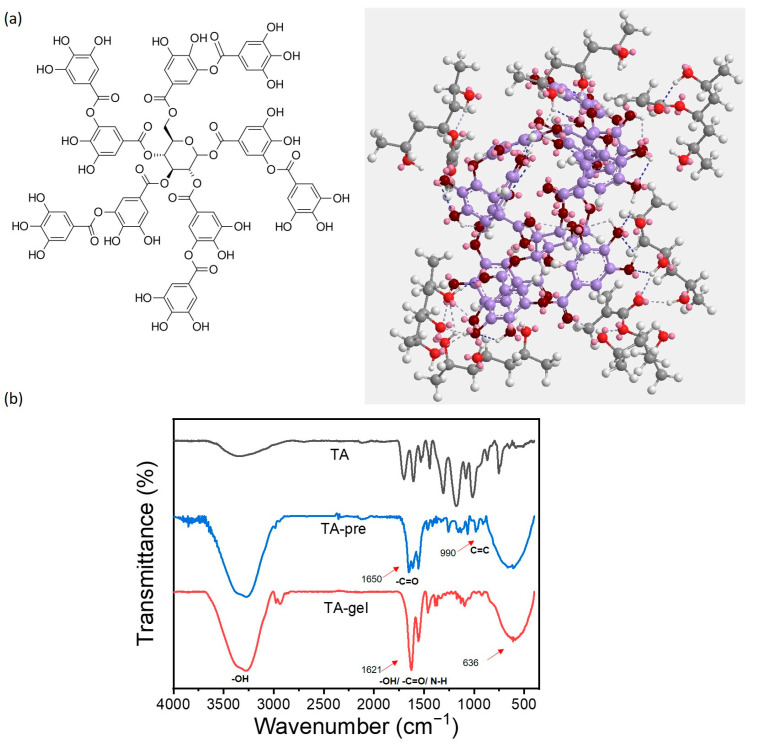
(**a**) Molecular structure of TA and conceivable interactions with PVA [47]. (**b**) FTIR spectrum of tannic acid (**top**), aqueous crosslinkable hydrogel precursor solution (**middle**), and resulting hydrogel (**bottom**).

The formation process of the PVA/TA/PVA-MA-g-PNIPAAm hydrogel is based on a former concept (Scheme 1). It relies on PVA functionalized with methacrylic acid (PVA-MA), giving a radically addressable vinylic side chain as grafting from point potentially leading to crosslinking. The triple network was generated from a mixture of PVA, PVA-MA, NiPAAm (N-isopropyl acrylamide), BIS (N,N′-methylene bis(acrylamide)), and tannic acid (TA) in distilled water (Figure 1: crosslinkable hydrogel precursor solution). The polymerization was triggered by radiation onto the photoinitiator (TPO-Li), leading to the graft copolymerization of NIPAAM onto PVA-MA, and the terpolymerization with BIS served to connect the grafts (Figure S1). Thus, a three-dimensional covalent polymer network was formed. The process ensures that the TA is molecularly distributed and largely immobilized in the network and provides further crosslinks by multiple hydrogen bonds to various moieties of PVA and/or PNiPAAm. The resulting gel products were then subjected to the typical freeze-thaw (F-T) method to induce nanocrystallization of PVA chain segments, which formed a third network that further promoted the mechanical strength of the hydrogel. The regular crystallization of PVA may be expected to take place in the presence of the applied small amounts of TA [45]. Note that although the crystallization of PVA is known to depend on the protocol used in the freeze-thaw procedure and the number of cycles, a variation in the conditions has not been carried out yet [48]. The importance of the F-T procedure for the properties of the crosslinked gels is to be mapped [49]. The resulting composite product constructed with a triple integration network was transparent, thermosensitive, and had self-repairing properties [43].

The polymerization of the olefinic entities in the presence of TA can be collaborated from the occurrence of aliphatic CH stretching vibrations at 2890 cm^−1^, and the loss of the C=C bending vibration at approximately 990 cm^−1^ (Figure 1b). The shift of the carbonyl stretching vibration from 1650 cm^−1^ to 1620 cm^−1^, overlapping with the O-H (of water and PVA in mutual interaction) and the N-H bending (of the amide moiety) vibrations, is the result of the polyreaction and the PVA crystallization [50,51]. 

**Figure 2:**
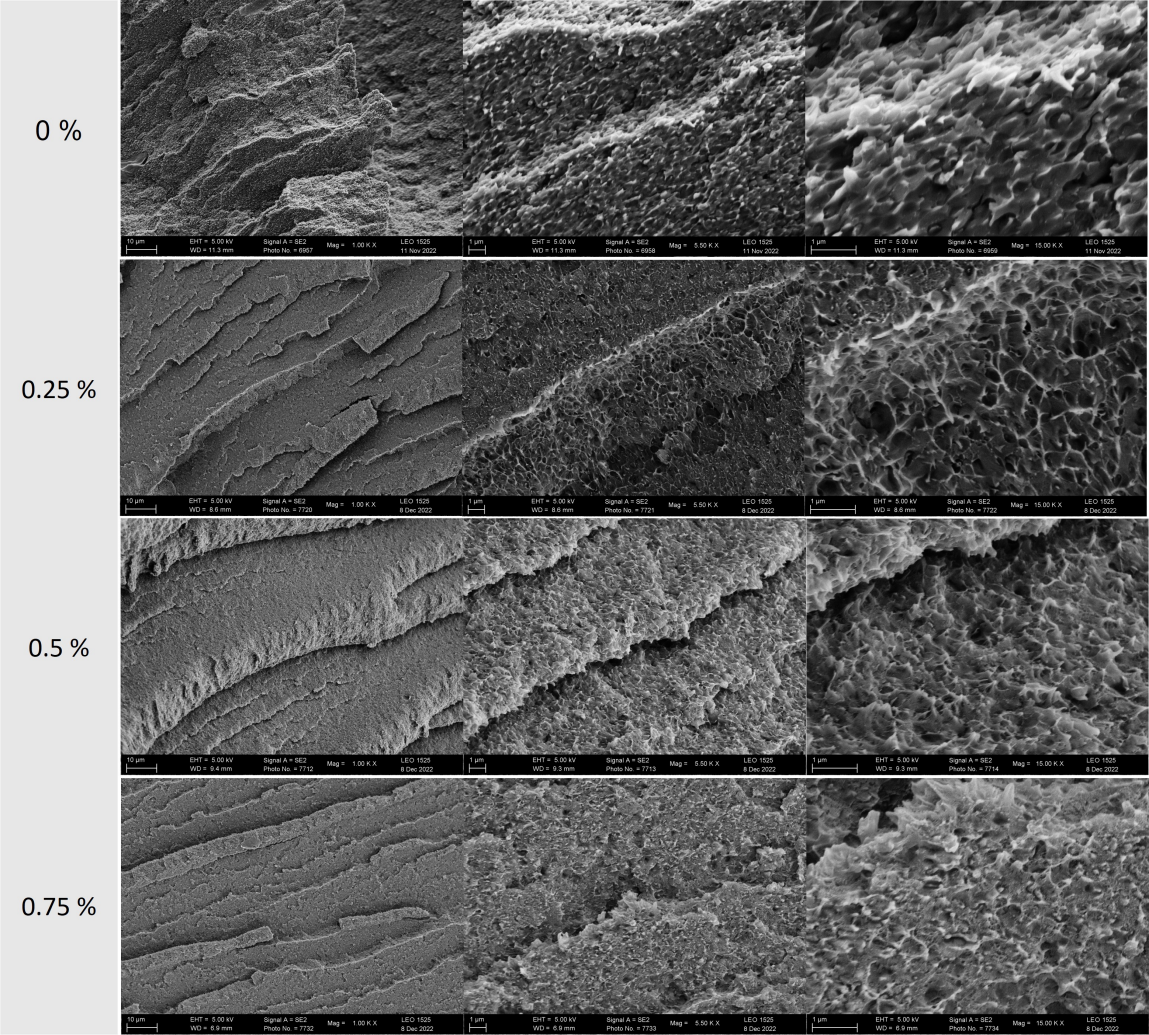
SEM images of the hydrogels with 0 wt.% TA, 0.25 wt.% TA, 0.5 wt.% TA, and 0.75 wt.% TA.

The hydrogels appear as three-dimensional foams after crystalizing the PVA in the freeze-thaw cycle, such as the hydrogel from PVA/PVA-MA with no tannic acid [23]. Only minor differences in morphology are observable, although the physical and mechanical properties are quite distinguishable. The gels become brittle after cooling in liquid nitrogen. The presence of TA causes the gel to fracture in definite layers, which is indicative of extended connectivity between the PVA crystals (Figure 2). The energy of the fracture is dispersed more extensively along the fracture surface into the sample. This was not observed for the gel without TA. In addition, the size of the macroscopic pores appears smaller at higher TA contents. TA seems to interfere with the growth of PVA crystals, thus smaller lamellae are formed that better fill the space. 

The XRD pattern and the thermal analysis by DSC of the gels are in line with the interpretation of the SEM images (Table 1) [52]. PVA crystallites as crosslinking points obtained by the F-T operation give rise to typical peaks in the XRD at angles of 19.6°, 22.9°, and 40.8° (Figure S2). The positions are more or less independent of the content of TA below 0.75 wt.%, which indicates that the presence of low amounts of TA does not change the PVA crystallization in a larger way. The width of the peaks does increase with the TA content, showing that the crystallites are indeed becoming smaller. An opposite trend is found for the melting point T_m_ for the same reason. The overall crystallinity is also somewhat lower at a higher TA content as the XRD peaks have a lower intensity, and the melting enthalpy ΔH_m_ is lower as well [53]. 

### 2.2. Mechanical Properties and Self-Healing Properties

**Figure 3:**
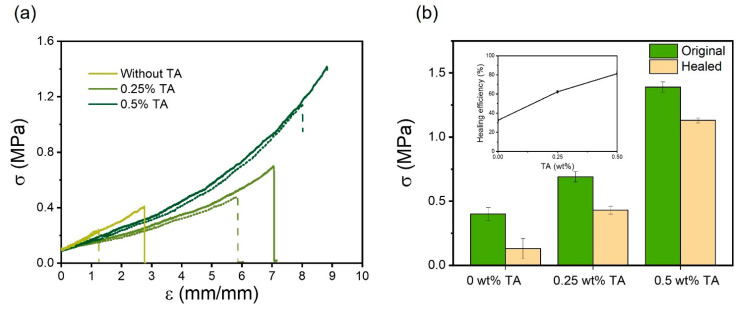
(**a**) Fracture tensile curves of the original (solid) and corresponding self-healed (dashed) triple-network hydrogels; (**b**) fracture tensile strength at break and the corresponding healing efficiency (inset).

The tensile strength (from uniaxial extension measurements) of the hydrogels increases monotonously with the TA content. The highest value is thus found for the gel with the highest studied content of 0.5 wt.% of TA (1.42 MPa), which reaches nearly 3.5 times the strength of the hydrogel without TA (0.4 MPa). An improvement is also found in the elongation at break, reaching up to 8.8, which compares favorably to the 1.2 for a gel without TA (Figure 3). The not-so-usual simultaneous improvement of the tensile strength and the elongation at break indicates that a further network contributes to the mechanical strength. This behavior may also be understood in terms of TA interfering with PVA crystallization. As mentioned above, the presence of TA seems to impede the formation of larger PVA crystallites, also leading to some more amorphous PVA. Such a gel would have a network that can be extended further before breaking and, concomitantly, can tolerate higher stress. In addition, the stiffness in terms of Young′s modulus is also moderately affected by the presence of TA; it first decreases and then increases again at a higher TA content (Figure 3a; Table S1). The decrease at 0.25 wt.% of TA may be also associated with the lower crystallinity. A longer arc length between the crystallites will translate into a smaller stiffness [54]. The increase at higher TA levels may indicate that additional interactions with TA and PVA increase chain segment stiffness, which overcomes the effects resulting from morphology changes.

The mechanical profile of the gel, of course, is the sum of the mutual influencing individual contributions of the conceivable three networks, with a prominent role for the various network crosslinks and their dynamics. The gel contains covalent BIS-based permanent bonds connecting two polymer chains and physical bonds connecting multiple chains in the form of PVA crystallites and hydrogen bonds to TAs. The dynamics of the physical interactions would be of obvious importance for self-healing after local failures [55], whereas the wide-meshed covalent crosslinks determine the long-range connectivity and, to a certain degree, also the shape. 

The chemical network can be disconnected by cutting the gel with a knife, and the self-healing properties can be assessed of the more dynamic parts of the gel (Figure S3). The extent of the self-healing ability of the network in a mechanical sense was evaluated after cutting the test specimen in the middle across the long axis and reuniting the parts directly after. Tensile testing after 2 h provided the same type of behavior, indicating that the original physical network was restored to a greater extent (Figure 3a, dashed lines; Figure 3b). The failure of the samples was always along the cutting plane, showing the importance of the additional chemical network to the overall integrity. The self-healing predominantly originates from the presence of the TA with regard tothe efficiency of reaching the original tensile strength at break. It follows the TA content (Figure 3b, inset). The PVA/PVA-MA-g-PNIPAAm hydrogel will restore approximately 30% of its original strength. The healing efficiency improved effectively to 81% for a gel with 0.5 wt.% of TA, notably to a level 3–4-times higher than that of an uncut gel without TA. The dynamics of noncovalent hydrogen bonding of PVA and, in particular, of TA are associated with self-healing [22]. The presence of TA could thus have a positive effect on damage to the integrity of the gel during swelling and deswelling cycles. 

### 2.3. Dynamics of the Network

**Figure 4:**
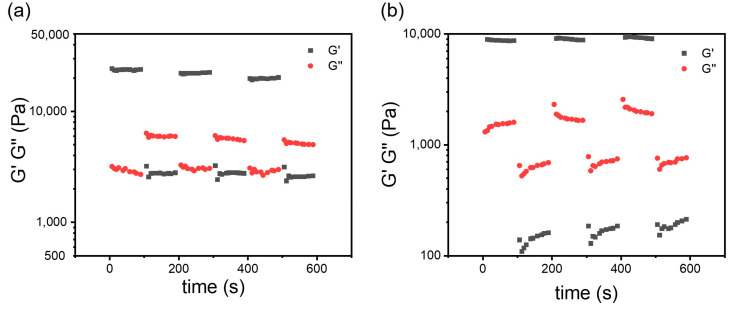
Alternate step strain sweep testing of 100 s duration between 1% and 250% at room temperature (1 Hz frequency) of (**a**) BIS-crosslinked PVA/TA (0.5 wt.%)/PVA-MA-g-PNIPAAm and (**b**) crosslinked PVA/TA (0.5 wt.%)/PVA-MA-g-PNIPAAm (Table S2).

The dynamics of the network making up the hydrogel with 0.5 wt.% of TA are below seconds, i.e., within the timeframe of an LCST transition. The dynamic behavior of this sufficiently responsive hydrogel was assessed using alternating steps of strain sweeps of 1% and 250% in a rheometer. An amplitude sweep indicates that the network is yielding approximately 100% in oscillatory mode at 1 Hz (Figure S3), indicative of a loss of its inner structure. This will be more extensive at 250% of strain, and a thinning is measurable immediately after applying such a strain. The storage moduli (G′) and loss moduli (G″) change reversibly within the time frame of the change in the sweep amplitude (Figure 4; Table S2). A reversible transformation between fluid- and gel-like was observed when changing the sweep amplitude from 250% to 1% and back. The values of the storage modulus G′ were almost an order of magnitude higher than those of the loss modulus G″ at small amplitude oscillatory shear (strain 1%), indicating the presence of an elastic gel. Application of the larger oscillatory shear strain of 250% leads more or less immediately to a loss of G′ below G″, a transition from a quasisolid state to a quasiliquid state. G″ increases in the process of liquefying, indicating that parts of the network have become mobile, adding to the resistance to flow. Returning to the smaller strain leads to re-establishing the gel properties. This procedure can be repeated several times with consecutive moderate losses of the G′ and G″ moduli relative to the starting values. The storage modulus, e.g., reaches a value of 87% of the initial value, and the loss modulus is 84% in the second cycle. These mechanical losses may originate from the adaption of the gel to the conditions of the measurement between the plates. The moduli are basically constant during the 100 s of observation, except for a small decrease (<10%). The exception is the second part of the first cycle, with a doubling of the storage module at 250% strain in 100 s to reach values that remain at ±3 MPa) in consecutive cycles. This may indicate that initial short arcs involving TA and PVA interaction in the amorphous part are replaced by longer ones, thus adding to the elasticity of the network. The half-life time for the buildup under strain is in the range of 15–20 s. 

The strain thinning and the return close to the starting values show the thixotropic properties of the gel and provide an indication of the dynamics of the processes to lie in the range of a second or below. The gel apparently loses its inner structure upon straining over 100% at a rate of 1 Hz. This must primary be correlated to the physical network of TA and of the amorphous PVA content. The covalent chemical network is providing a kind of shape-memory frame, which is stressed in the measurements, but allows the gel to return to its initial state, but not completely. Possibly, short arcs involving BIS are also permanently lost.

An analogous noncovalently crosslinked PVA/TA (0.5 wt.%)/PVA-MA-g-PNIPAAm hydrogel (prepared by omitting BIS from the recipe), a double network gel, has storage and loss moduli that are approximately a factor of three lower at 1% strain amplitude. Steps of strain sweeps show similar but not equal behavior (Figure 4b). The double network of crystalline PVA and TA hydrogen bonds shows the same thixotropy with a fast break up and restoration of the inner structure upon straining at 250% amplitude, respectively, upon returning to a strain of 1%. Now it is observed that a substantial reorganization takes place during the 100 s of observation in the first two cycles. The half-life of the changes during the 100 s again is in the range of 20 s. It leads to a small overall increase in storage and loss moduli between cycles at 1% of strain, and a further decline at 250%. The extent of the adaption to the conditions of the step sweep experiments is obviously more extensive than for the triple network involving BIS. The chemical network thus seems important for shape memory, with a faster return to the most stable gel structure. 

### 2.4. Equilibrium Swelling

**Figure 5:**
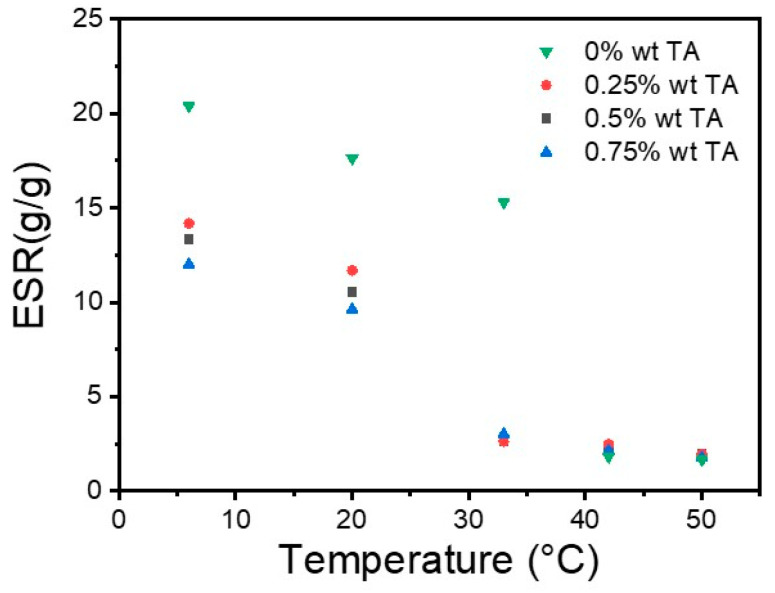
Temperature dependence of the equilibrium swelling ratio (ESR) of the triple-network hydrogels with different content of TA over the temperature range from 6 to 50 °C.

A typical thermoresponsive profile for the swelling ratio is obtained in water in the temperature range between 6 °C and 50 °C: The temperature equilibrium swelling ratio of the hydrogels with TA shows the expected transition is approximately 30 °C for a polymer with an LCST (Figure 5). The swelling ratio of the hydrogel with 0.25 wt.% TA (13.3 to 2.0) is lower than the system without TA (18.6 to 1.2), and generally the swelling ratio is lower at a higher content of TA (from 0.25 wt.% to 0.75 wt.%). This trend is opposite to that found for the mechanical properties, indicating that TA indeed is involved in the formation of a further network that also impacts the swelling. The phase transition is found at lower temperatures with a higher TA content. This may relate to TA interacting with PVA and NIPAAm hydrogen bond-forming entities, decreasing the critical solution temperature. The degree of hydrolysis of polyvinyl acetate is known to affect the thermal properties, such as the presence of hydrogen bond-forming entities or a change in the ionic strength [56].

### 2.5. Dynamic Swelling Properties

**Figure 6:**
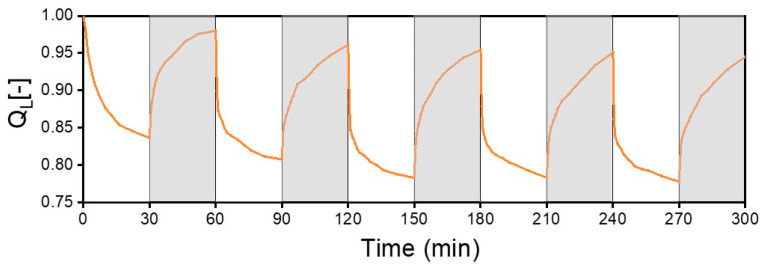
Length normalized swelling degrees (QL) of triple-network hydrogels (0.5 wt.% TA). Exemplary progress of swell degrees under cyclic switching between 40 °C and room temperature (grey shading) in deionized water.

**Figure 7:**
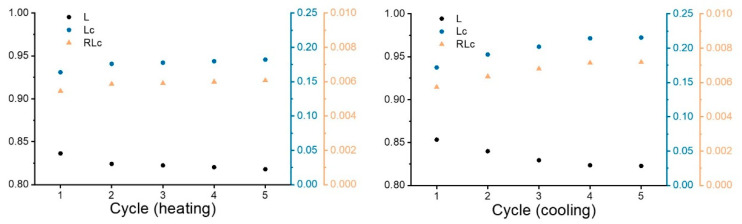
Length (L), final length change (LC), and its linear rate constant (RLC) of a PVA/TA/PVA-MA-g-PNIPAAm triple-network hydrogel cycled in 30 min intervals between 20 °C and 40 °C in deionized water.

The interest in the gel in this study concerned the kinetics of the swelling-deswelling process and the recovery of the gel after deswelling. The target was a gel with approximately constant dimensions for use as a thermoresponsive valve [20]. The dynamic swelling at 40 °C and deswelling at 25 °C were monitored with a camera during five consecutive cycles by placing disk-shaped gels alternately from a bath of the lower temperature to one with the higher temperature and vice versa. The dimensions of the gel were normalized to the equilibrium degree of swelling at 25 °C (Table S3). The gel reacts sensitively to the temperature change; the dynamics are the highest for the deswelling (Figure 6 and Figure 7): The majority of the volume loss occurs within 5 min, after which the process becomes much slower. The rehydration at 25 °C also shows a process with several time constants. Additionally, here, a fast volume change (increase) is followed by a slower secondary increase (Table 2). Processes with at least two time constants were observed before for this system and were associated with the different types of interactions in the interpenetrating network. 

The maximum diameter after reswelling decreases in the first three cycles (Figure 7). Relatively constant dimensions of the swollen and de-swollen states are reached after that. Indeed, the dimensional changes between cycles monotonously get smaller with the number of cycles reaching some kind of final (equilibrium) shape in both the swollen and de-swollen states. 

Apparently, again, some reorganization of the interactions formed in the synthesis of the gel takes place. Part of the water would be irreversibly lost at the beginning of the de-swelling swelling cycles, possibly with a substitution by the strong potential H-bonding sites provided by TA, increasing the efficiency of reconstruction, and finally dynamically leading to a balance between loss and gain of water. The restructuring of the gel is also reflected in the rate of deswelling and deswelling (Figure 7). The presence of a highly dynamic third network within the gel, with the potential for self-healing and some shape memory, obviously, may be used to reach a state with relatively constant dimensions in the swollen and collapsed state. These are exactly the properties useful for a robust thermal valve with an extended service life. Currently, investigations are carried out to validate the concept in a device and in contact with reagents.

## 3. Conclusions

A novel triple-network hydrogel possessing good toughness, smart functional behavior of thermoresponsive, and self-healing has been successfully synthesized by a simple one-step polymerization and F-T operation. The prepared triple-network hydrogel exhibits better mechanical properties than the corresponding double-network hydrogel [21]. The “virgin” thermosensitivity is diminished slightly (water capacity: 87% vs. 84%) and reaches a steady state after a couple of cycles. The hydrogel also shows substantial self-healing properties with 81% of healing efficiency, which may underlie the robust and reversible swelling-deswelling behavior. The self-healing provides the opportunity to “repair” the gel operating as a valve in a chemical reactor, simply by adding a small amount of particulate gel. The latter will integrate itself into the valve. The described design reveals a broader potential in abundant areas such as actuators, components of smart devices, and so on.

## 4. Materials and Methods

### 4.1. Materials

Poly(vinyl alcohol) (PVA, 99+% hydrolyzed PVA, Mw 85,000–124,000), hydroquinone, N,N′-methylenebis(acrylamide) 99%, lithium phenyl-2,4,6-trimethyl-benzolphosphonate (TPO-Li) ≥95%, triethyl amine 99%, tannic acid (99%) were purchased from Sigma-Aldrich (Taufkirchen, Germany). N-isopropyl acrylamide (NIPAM, 99%, pure, stabilized) was obtained from Acros Organic (Darmstadt, Germany). Methacrylic acid (≥99%) was provided by Merck KGaA (Darmstadt, Germany). All reagents were used as received. Demineralized water (DI, <0.1 μS·cm^−1^) was obtained from lab water systems.

### 4.2. Synthesis of PVA-MA

PVA-MA was synthesized by the method of our previous study [21]. PVA (15 g) was dissolved in 135 mL of deionized water in a round flask equipped with a condenser at 90 °C by stirring for 1 h. After the solution was cooled to room temperature, 30 mg of hydroquinone, 20 mL of MA, and 7.5 mL of hydrochloric acid (0.5 M) were added to the solution. The resulting mixture was then stirred under 60 °C for 12 h to complete the esterification. After cooling, 0.5 mL of triethylamine was added to reach a neutral pH. The resulting solution was then diluted to 1.5 L with water and then precipitated in acetone (1.5 L). The precipitate was filtered, washed, and dried at 60 °C in a vacuum oven (1000 mbar).

### 4.3. Hydrogel Preparation

A 5 wt.% of PVA and a 5 wt.% of PVA-MA were dissolved in deionized water in a round flask with a stirring bar by heating at 90 °C under stirring for 1 h. Tannic acid (1 g) was dissolved in deionized water (1 mL) at 50 °C. Different quantities of tannic acid solutions were added under stirring. After the solution was cooled down to room temperature, 20 wt.% of monomer NIPAM was added to the solution, which was stirred until it presented a uniform state. Subsequently, 0.1 wt.% of TPO-Li as photo-initiator and 0.2 wt.% of N, N’-methylene bisacrylamide (BIS) as a crosslinking agent were added. The mixture was immersed into an ultrasonic bath at room temperature for over 30 min to remove dissolved components of air. The precursor solution was syringed into different shapes (for multi-tests) of PLA molds formed by fused deposition method (FDM) using a 3D printer (Ultimaker 2, Ultimaker B.V., New York, NY 10041, USA). The precursor was exposed to a high-power (60 W output) UV light (405 nm, Geeetech, Shenzhen, China) in an ice bath for 10 min. The attained hydrogel was then removed from the mold, soaked in DI water for 24 h to extract soluble components (residual monomers, etc.). The final hydrogels were formed through freezing-thawing procedure (freezing at −32 °C over 12 h, thawing at 25 °C for 2 h). The resulting hydrogels were kept in deionized water for further characterization and analysis.

### 4.4. Characterization

#### 4.4.1. FT-IR Characterization

The hydrogel samples were dried overnight in a vacuum oven at 60 °C to remove moisture and were then crushed to a powder. Attenuated total reflectance-Fourier transform infrared (ATR-FTIR) spectra were recorded using Vertex 70 (BRUKER OPTIK GmbH, Ettlingen, Germany), ranging of 4000–400 cm^−1^ with a 2 cm^−1^ resolution and 32 scans.

#### 4.4.2. Equilibrium Swelling

The hydrophilicity of the hydrogel was studied via a swelling equilibrium experiment in the temperature range of 6 °C to 50 °C. The equilibrium swelling ratio (ESR= Ws/Wd × 100%) is defined as the weight of water-swollen state (Ws) divided by the weight of the dehydrated state (Wd). Hydrogel samples were prepared in a 3D-printed circular model (3 cm in diameter), and immersed in deionized water for 12 h to reach the equilibrium state. Then the samples were weighed after wiping off the residual water on the surface. Subsequently, the samples were placed in a vacuum drier (1000 mbar) at 60 °C overnight for dehydration, and then the weight of the dehydrated state was recorded.

#### 4.4.3. Length Normalized Swelling Degrees

The aforementioned circular samples were firstly immersed in deionized water to reach an equilibrium state. Temperature of a water bath was switched from low (room temperature) to higher (over LCST), and a video of the volume change was recorded using a camera. Imagine-Pro Plus 6.0 software (Media Cybernetics, Rockville, MD, USA) was employed for the analysis of the dimensional changes. Length-normalized swelling degrees Q_L_ were calculated using the equation: $Q_{L}=\frac{L_{S}}{L_{Q}}*100\%$, where Ls is the length of the swollen hydrogel and Lq is the length of the equilibrium hydrogel.

#### 4.4.4. Scanning Electron Microscopy (SEM)

The hydrogel samples were fractured after cooling in liquid nitrogen for 10 min, and then immediately submitted to a freeze dryer (Gamma 2-16 LSCplus, Osterode am Harz, Germany) and lyophilized over 24 h. All the samples were coated with a thin layer of gold by sputtering. The surface morphology was evaluated using a LEO 1525 Field Emission Scanning Electron Microscope (LEO Electron Microscopy Inc., One Zeiss Drive, Thornwood, NY 10594, USA) under 5 kV accelerating voltage.

#### 4.4.5. Mechanical Properties

The tensile testing was carried out on a Zwick Z2.5 universal testing machine (Zwick/Roell, Ulm, Germany) according to ASTM D412. The dumbbell-shaped specimens (width 4 mm, thickness 2 mm, length 25 mm) were prepared in a corresponding 3D-printed model. The original samples were cut in half and then horizontally placed together at room temperature for 2 h in self-healing experiments. The samples were coated with silicone oil to avoid excessive dehydration, and then clamped on both sides and stretched at a constant rate of 5 mm/min. The fracture stress and strain were recorded from stress-strain curves at the breaking point.

#### 4.4.6. Rheological Measurements

The self-healing properties of the prepared hydrogels were evaluated in a rotary rheometer (AR G2, Newcastle, Germany) using plate-and-plate geometry (diameter 20 mm, gap 1000 um). The hydrogel samples were subjected to dynamic rheological experiments; amplitude oscillatory strain circles from small strains (γ = 1%) to subsequent large strains (γ = 250%) were operated in every 100 s before every strain interval. The time dependence of storage modulus and loss modulus was recorded in continuous measurement at a constant frequency (1 Hz) at room temperature.

## Figures and Tables

**Table 1:** Physical properties of PVA/TA/PVA-MA-g-PNIPAAm hydrogels.

TA content (wt.%)	0	0.25	0.50
XRD (peak at 19.6°)			
Integral value	74,352	69,373	67,688
peak	6922	6583	6049
FWHM * (°)	8.9	11.2	15.1
DSC			
T_m_ (°C)	226.5	223.8	221.8
ΔH_m_ (J/g)	22.3 ± 0.3	21.6 ± 0.2	20.1 ± 0.1
crystallinity (%)	16.3 ± 0.2	15.7 ± 0.2	14.6 ± 0.1

*: Full width at half peak height

**Table 2:** The time constant (min^−1^) of the last deswelling-swelling loop.

Time (min)	240–245	280–300
Deswelling	−0.0072	−0.0013
Swelling	0.0092	0.0029

## Data Availability

The datasets generated and analyzed for this study can be shared upon reasonable request from the corresponding author.

## Supplementary Materials

The following supporting information can be downloaded at: https://www.mdpi.com/article/10.3390/gels9090695/s1, Figure S1: Photos of the hydrogel in each fabrication progress; Figure S2: XRD spectra of the hydrogels without TA (black), with 0.25 wt.% TA (red) and 0.5 wt.% TA (blue); Figure S3: The mechanism of self-healing; Figure S4: The rheological properties of hydrogels: the amplitude sweeps (a) and the frequency sweeps (b) of the hydrogels; Table S1: Mechanical data of PVA/TA(0.5 wt.%)/PVA-MA-g-PNIPAAm hydrogel at 25 °C; Table S2: Rheological data of BIS-crosslinked PVA/TA (0.5 wt.%)/PVA-MA-g-PNIPAAm and crosslinked PVA/TA(0.5 wt.%)/PVA-MA-g-PNIPAAm; Table S3: Length (L), final length change (LC), and its linear rate constant (RLC) of PVA/TA(0.5 wt.%)/PVA-MA-g-PNIPAAm, while cycling in 30 min intervals between 25 °C and 50 °C in deionized water; Movie S1: Stretch test for self-healed hydrogels.
