# Supplementary material for: Tough, Stretchable, and Thermoresponsive Smart Hydrogels
Source: Gels. 2023 Aug 28;9(9):695. doi: 10.3390/gels9090695 (PMC10528277; doi:10.3390/gels9090695)
Supplement: Supplementary file 1 [file gels-09-00695-s001.zip › gels-2507919-supplementary.pdf]

gels

Supplementary Materials

Fabrication of Thermo-Responsive Controllable Shape-Changing Hydrogel

Yi Luo, Werner Pauer and Gerrit A. Luinstra *

* Correspondence: luinstra@chemie.uni-hamburg.de;

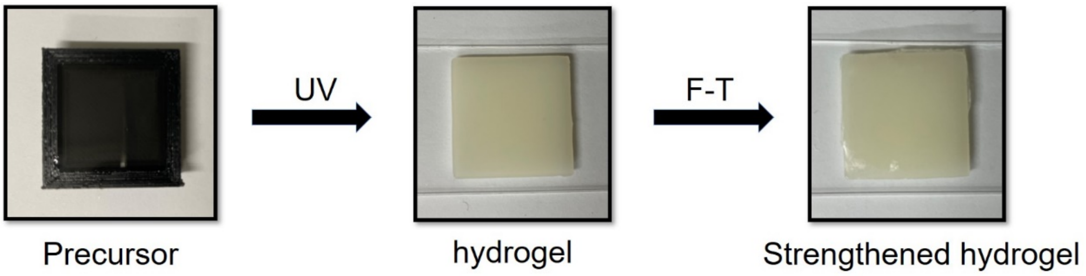

Figure S1. Photos of the hydrogel in each fabrication progress.

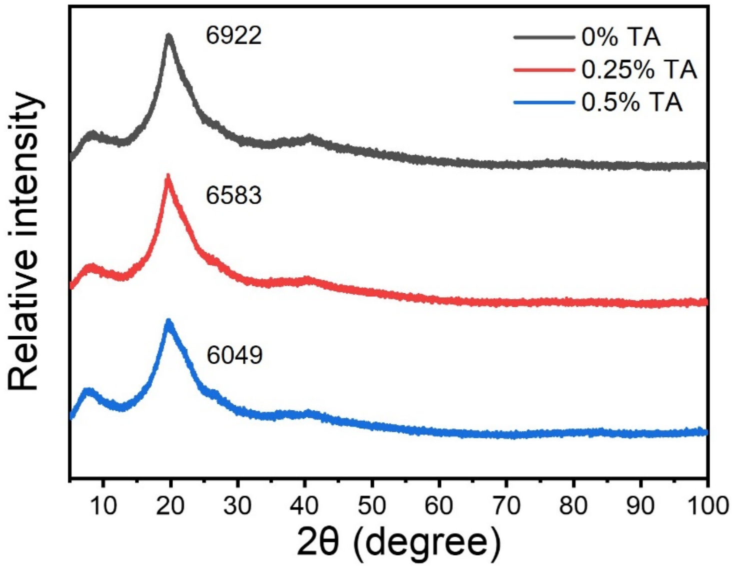

Figure S2. XRD spectra of the hydrogel without TA (black), with 0.25 wt.% TA (red) and 0.5 wt.% TA (blue).

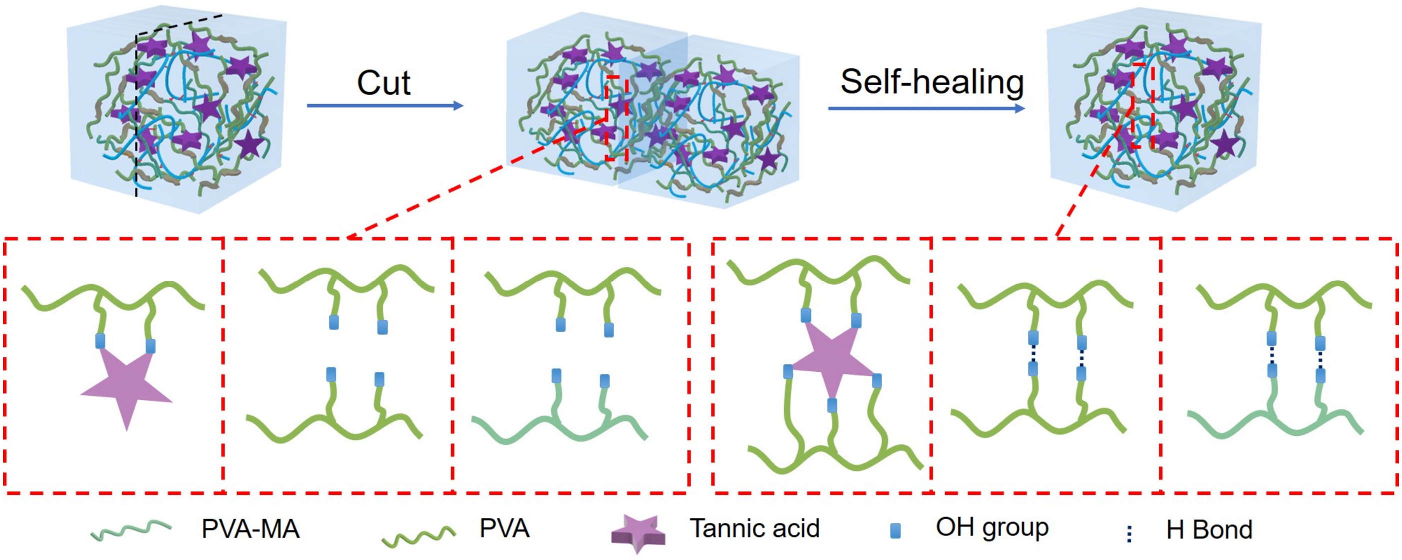

Figure S3. The mechanism of the self-healing.

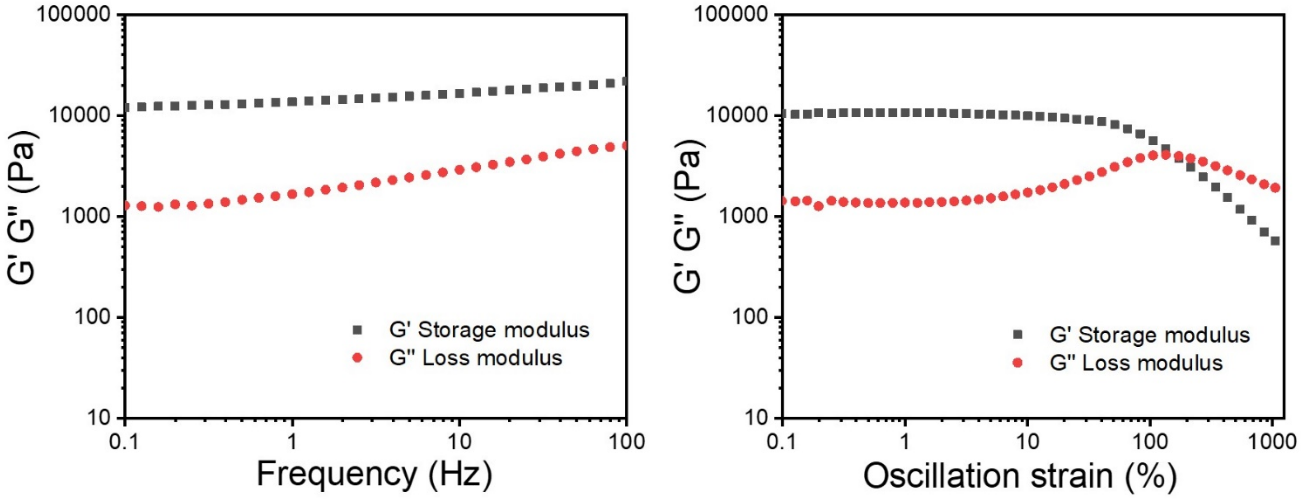

Figure S4. The rheological properties of hydrogels: the amplitude sweeps (a) and the frequency sweeps (b) of the hydrogels.

Table S1. Mechanical data of PVA/TA(0.5 wt.%)/PVA-MA-g-PNIPAAm hydrogel at 25°C

TA content (wt)	0%		0.25%		0.50%	
	original	self-healed	original	self-healed	original	self-healed
Young's modulus (MPa)	0.12	0.108	0.058	0.044	0.085	0.082
elongation at break (mm/mm)	2.76	1.24	7.05	5.87	8.82	8
stress at break (MPa)	0.4 (0.05)	0.13 (0.08)	0.69 (0.04)	0.43 (0.03)	1.39 (0.04)	1.13 (0.02)
healing-efficiency HE (%)	33		62		81	

Table S2. Rheological data of BIS-crosslinked PVA/TA (0.5 wt.%)/PVA-MA-g-PNIPAAm and crosslinked PVA/TA(0.5 wt.%)/PVA-MA-g-PNIPAAm.

Time (s)	Oscillatory stress (Pa)		G′ (Pa)		G′′ (Pa)	
	BIS-crosslinked	Physically crosslinked	BIS-crosslinked	Physically crosslinked	BIS-crosslinked	Physically crosslinked
6	89.36	11.61	24390	10510	3191	1307
13	241.1	95.44	23630	8930	3058	1344
19	237.2	88.87	23290	8862	3002	1453
26	235.3	88.46	23720	8812	3099	1467
39	237	88.01	23850	8771	2927	1534
45	238.3	87.94	23910	8755	3047	1520
58	236.3	87.62	23830	8703	2861	1550
70	235.4	87.36	23380	8714	2847	1551
77	236	87.33	23730	8660	2770	1574
90	237.2	87.17	23920	8684	2705	1595
106	9827	1334	1204	138.9	6363	649.8
113	11730	1379	1459	109.5	5846	524.2
119	12760	1389	1759	117.4	6074	548.9
126	13680	1458	1974	125.5	6008	576.1
139	14950	1584	2272	142.4	5962	621.9
145	15350	1595	2378	144.1	5973	623.3
158	15890	1663	2528	151	5892	650.9
170	16150	1685	2663	154.3	5959	659.2
177	16320	1727	2732	159	5998	672.3
190	16380	1770	2793	161.4	5953	690.1
206	87.08	11.93	22140	11800	3278	2313
213	221.6	102.6	21710	9081	3142	1888
219	219.9	91.94	22030	9174	3203	1831
226	222.1	92.06	22060	9127	3048	1773
239	223.5	91.33	22150	9054	3008	1740
245	223.4	90.99	22130	9033	2914	1712
258	223.9	90.2	22120	8937	3064	1700
270	224.7	89.56	22350	8884	3091	1676

277	224.9	89.1	22340	8825	3003	1659
290	225.7	88.59	22470	8800	3060	1663
306	12910	1517	3238	185.5	6048	781.5
313	16430	1557	2421	129.2	5601	583
319	15470	1630	2745	149.9	5803	649.8
326	15940	1636	2709	147.2	5757	636.9
339	15870	1715	2774	159.3	5738	674.1
345	15860	1782	2794	168.3	5723	699
358	15760	1814	2801	172.3	5658	706.5
370	15590	1834	2797	175.5	5587	715.6
377	15490	1844	2778	177.1	5531	718.9
390	15230	1915	2760	185.1	5455	745.9
406	83.46	11.73	19850	12150	3084	2567
413	198.1	106	19270	9308	2790	2182
419	195.7	95.37	19660	9489	3002	2183
426	198.1	95.73	19640	9439	2849	2106
439	199.7	94.88	19880	9366	2838	2056
445	199.8	94.31	19780	9308	2654	2005
458	199.9	93.51	19680	9248	2808	1987
470	200.2	92.66	19980	9177	2945	1945
477	201.4	92.44	19970	9121	2911	1945
490	203.1	91.49	20240	9054	2994	1908
506	12160	1613	3151	190.8	5537	757.2
513	15230	1697	2360	153.3	5142	601.6
519	14270	1708	2613	175.2	5269	657.6
526	14570	1795	2554	182.8	5213	677.4
539	14470	1811	2582	175.45	5189	694.1
545	14390	1860	2578	178.75	5156	689.9
558	14220	1868	2578	191.2	5085	698.6
570	14160	1874	2577	199.25	5054	744.9
577	14120	1916	2607	205.8	5038	748.2
590	14110	2070	2624	212.3	5016	764.4

Table S3. Length (L). final length change (LC). and its linear rate constant (R_{LC}) of PVA/TA(0.5 wt%)/PVA-MA-g-PNIPAAm while cycling in 30 min intervals between 25 °C and 50 °C in deionized water.

operation	L (%)	LC (%)	R_{LC} (%/min)
heat 1	83.63	16.37	16.37
cool 1	85.34	14.37	17.18
heat 2	82.40	17.25	17.60
cool 2	83.99	15.40	19.07
heat 3	82.23	17.20	17.77
cool 3	82.93	16.16	20.21
heat 4	82.03	17.16	17.97
cool 4	82.35	16.78	21.43
heat 5	81.79	17.31	18.21
cool 5	82.27	16.76	21.55
